# Exploring the pathway: clinical utility and open challenges of targeting BRAF alterations in biliary tract cancers and gastrointestinal malignancies

**DOI:** 10.1016/j.esmogo.2024.100129

**Published:** 2025-01-14

**Authors:** L. Weiss, D. Zhang, W.G. Kunz, S. Boeck, G. Curigliano, V. Subbiah, F. Lordick, T. Brummer, C.B. Westphalen, L. Boscolo Bielo

**Affiliations:** 1 Department of Medicine III, LMU Klinikum, University of Munich, Munich, Germany; 2 German Cancer Consortium (DKTK) Partner Site Munich, German Cancer Research Center (DKFZ), Heidelberg, Germany; 3 Department of Radiology, University Hospital, LMU Munich, Munich, Germany; 4 Department of Hematology and Oncology, München Klinik Neuperlach, Munich, Germany; 5 Early Drug Development for Innovative Therapies, European Institute of Oncology IRCCS, Milan, Italy; 6 Department of Oncology and Hemato-Oncology, University of Milan, Milan, Italy; 7 Sarah Cannon Research Institute, Nashville, USA; 8 Department of Medicine, Division of Oncology, University Cancer Center (UCCL), University Hospital Leipzig, Leipzig, Germany; 9 Institute of Molecular Medicine and Cell Research (IMMZ), Faculty of Medicine, University of Freiburg, Freiburg, Germany; 10 German Cancer Consortium (DKTK) Partner Site Freiburg, German Cancer Research Center (DKFZ), Heidelberg, Germany; 11 Comprehensive Cancer Centre Munich, LMU Klinikum, University of Munich, Munich, Germany

**Keywords:** biliary tract cancers, BRAF, BRAF V600E, gastrointestinal cancers, precision oncology, targeted therapy

## Abstract

The *BRAF* proto-oncogene plays a key role in oncogenesis, promoting growth and survival through various genetic alterations. Most mutations involve the substitution of valine at amino acid position 600 (V600), resulting in a constitutively active protein, known as class I alterations. The most common class I mutation encodes for the BRAF^V600E^ oncoprotein. Therapeutic targeting of BRAF^V600E^ has led to the approval of dabrafenib plus trametinib for solid tumours [excluding colorectal cancer (CRC)] refractory to standard therapies. In gastrointestinal cancers, dabrafenib and trametinib have shown remarkable results in biliary tract cancers (BTC), establishing this combination as a viable second-line option for *BRAF*^V600E^-mutant BTC. In CRC, intrinsic epidermal growth factor receptor (EGFR) activation circumvents BRAF inhibition, necessitating the concurrent use of EGFR inhibitors, whose treatment strategy led to the approval of encorafenib plus cetuximab for *BRAF*^V600E^ CRC.

Despite these advances, resistance to BRAF inhibitors is almost universal, often due to extracellular signal-regulated kinase (ERK)-mediated up-regulation of RAF dimers that is able to overcome the inhibition of BRAF monomers. This resistance mechanism has spurred the development of novel RAF inhibitors able to prevent or to inhibit RAF dimers. Additionally, several treatment strategies are currently being investigated, including multi-step vertical mitogen-activated protein kinase (MAPK) inhibition and targeting parallel signalling pathways capable of bypassing MAPK oncogenic inhibition. While noteworthy results have been achieved with BRAF^V600E^ inhibition, further research is needed to optimize BRAF-targeted therapies and address resistance mechanisms. Continued research and innovation are crucial to improving patient outcomes and addressing the complexities of *BRAF* mutations in human cancers.

## Highlights

• BRAF^V600E^ targeting shows promise in biliary tract and gastrointestinal cancers.
• Combination of dabrafenib and trametinib is effective for BRAF^V600E^ biliary cancers.
• Resistance to BRAF inhibitors often arises due to ERK-mediated RAF dimer formation.
• Novel RAF inhibitors may overcome resistance by blocking RAF dimerization.
• Targeted therapies in BTC show improved outcomes for BRAF^V600E^ mutations.

## Introduction

Biliary tract cancers (BTC) include a heterogeneous group of aggressive malignancies, representing about 1% of all solid cancers and 10%-15% of all primary liver tumours.^1^^,^^2^ Despite the improved outcomes achieved with the recent addition of immunotherapy to front-line chemotherapy regimens, median overall survival (OS) for patients with advanced BTC still approximates only 1 year,^3^^,^^4^ with very limited benefits achieved from currently available treatment regimens beyond the first line.^5^ Considering their dismal prognoses, research has focused on detecting additional biomarkers to gather further treatment opportunities, ultimately leading to the definition of multiple relevant therapeutic targets, particularly enriched among intrahepatic BTC.^6^ Accordingly, international guidelines have endorsed the routine implementation of next-generation sequencing (NGS) for patients with advanced BTC in order to identify tumours exhibiting actionable alterations with available approved treatment options.^2^^,^^7^

Among recurrent alterations, mutations involving the BRAF proto-oncogene (*BRAF*^MUT^) are found in ∼5%-9% of BTC,^6^^,^^8^^,^^9^ occurring almost exclusively in intrahepatic BTC. Importantly, BRAF^MUT^ confers a distinct clinical profile to BTC, associated with a more advanced stage at the time of diagnosis, an aggressive clinical course, and worse OS with reduced benefits from conventional cytotoxic chemotherapies.^10^^,^^11^

BRAF encodes for a serine/threonine kinase located within the mitogen-activated protein kinase (MAPK) pathway, which is dysregulated in about 7%-15% of all tumours.^12^, ^13^, ^14^ Several molecular alterations have been described to alter the *BRAF* proto-oncogene, with most consisting of a substitution of valine at position 600 (V600), leading to a constitutively active conformation of the protein^15^ capable of promoting proliferation, migration, and survival of tumour cells.^16^

Notably, targeting BRAF^V600E^ has led in 2022 to the tumour-agnostic Food and Drug Administration (FDA) approval of the BRAF inhibitor dabrafenib in combination with trametinib, an allosteric MEK inhibitor, for solid tumours carrying BRAF^V600E^. This approval was granted based on the results of multiple cohort trials including 131 adult and 36 paediatric patients, in which the combination of dabrafenib and trametinib showed remarkable and consistent efficacy across a wide range of solid tumours,^17^, ^18^, ^19^ and was supported by the results of the COMBI-d, COMBI-v, and BRF113928 studies,^20^^,^^21^ leading to the establishment of BRAF^V600E^ as a tier I biomarker according to the European Society for Medical Oncology (ESMO) Scale for Clinical Actionability of Molecular Targets (ESCAT).^2^^,^^7^^,^^22^

Accordingly, the present work, leveraging on a case report showing prolonged clinical benefits from the use of dabrafenib plus trametinib, will outline the clinical utility achieved by targeting BRAF^V600E^ among BTC and gastrointestinal cancers. In addition, it will address the clinical challenges still existent in targeting the oncogenic MAPK signalling cascade and will discuss treatment strategies under development aimed to further tackle the pathway to achieve better clinical outcomes for patients affected by BRAF^MUT^ tumours.

## Case report

### Diagnosis and initial stage

The presented case involves a patient with intrahepatic BTC harbouring a *BRAF*^V600E^ mutation.

This study was conducted with the principles of the Declaration of Helsinki and with the guideline for good clinical practice. The study was approved by the ethic committee of the Medical Faculty of the Ludwig-Maximilians-University Munich (Reference number: 21-0869).

### Initial treatment and course

In April 2017, the diagnosis of intrahepatic cholangiocarcinoma was made and the patient underwent anatomical central liver resection of segments IVa/b and V, as well as an open cholecystectomy. Additionally, lymph node dissection of the hepatoduodenal ligament was carried out. The post-operative tumour classification according to UICC 2017 was pT1b, pN0 stage IB. Following surgery, a structured follow-up was initiated.

In December 2017, three new intrahepatic lesions were detected, with radiological features indicating BTC recurrence. After an interdisciplinary case discussion in the gastrointestinal tumour board, systemic therapy with gemcitabine/cisplatin was initiated.

### Therapy course

After three cycles of gemcitabine and cisplatin, magnetic resonance imaging (MRI) staging showed locoregional tumour progression. The gastrointestinal tumour board recommended brachytherapy since all lesions were accessible for local ablation. In May 2018, the patient received brachytherapy for four liver lesions in the right lobe and HDR brachytherapy for lesions in segments III and V. The ablated metastases remained stable until January 2019, until multiple new hepatic metastases emerged during follow-up.

In February 2019, systemic therapy with FOLFOX was initiated, achieving disease stabilization. However, by April 2020, disease progression eventually occurred, prompting further brachytherapies (segment VI and VIII in April 2020, segment I and II in May 2020).

### Extended molecular diagnostics and targeted therapy

**Figure 1:**
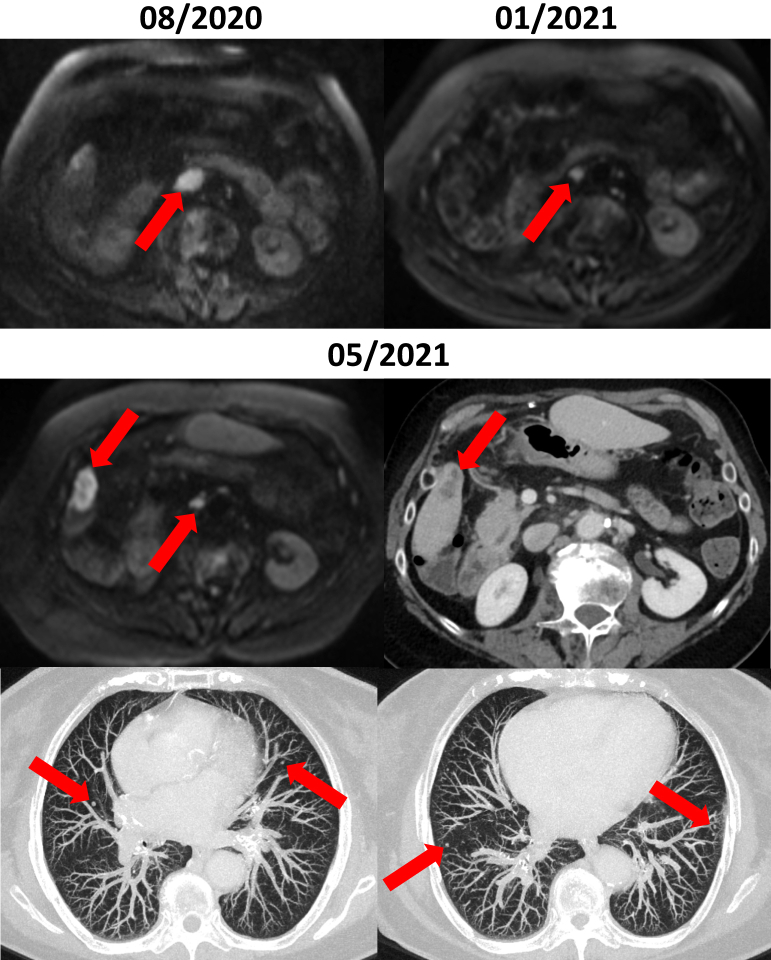
**Baseline target lesions and radiological response to dabrafenib and trametinib.** The top left image shows the nodal tumour burden at baseline before start of treatment with a large retroperitoneal lymph node metastasis. The top right image depicts the tumour regression after systemic treatment with complete remission of the lymph node metastasis. The images of the second and third row demonstrate the tumour relapse with a new hepatic metastasis in segment 5 and multiple new lung metastases.

Comprehensive genomic profiling using the amplicon-based, Ion Torrent-based Oncomine Focus Assay (Thermo Fisher Scientific, Waltham, MA)^23^ was carried out on a new liver biopsy detected a clonal *BRAF*^V600E^ mutation (allele frequency: 28.02%). The case was discussed in the institutional molecular tumour board at the University of Munich and *BRAF*-directed treatment was recommended. After securing cost coverage by the insurance provider, treatment with dabrafenib in combination with trametinib was initiated in December 2020. The targeted therapy was well tolerated. In March 2021, liver MRI showed significant response and subsequent radiological evaluations confirmed disease stabilization (June 2021, September 2021, January 2022) (Figure 1). After 18 months of treatment, hepatic, lung, and interaortocaval lymph node progression occurred.

### Further treatments

After progression on dabrafenib/trametinib, the patient received six cycles of dose-reduced FOLFOX. Due to significant peripheral polyneuropathy, treatment was de-escalated to folinic acid in combination with 5-fluorouracil for 16 cycles. Microsatellite status was determined as stable (MSS) during disease course in 2018 and programmed death-ligand 1 status was tested negative in 2022 (tumour proportion score 0%, combined positive score 0, immune cell score 0).

Based on the DART study, a cost coverage application for dual checkpoint inhibition with nivolumab/ipilimumab was submitted to the insurance provider and granted access in September 2023.^24^^,^^25^ After disease progression under FOLFIRI (received from June 2023 to August 2023), the patient received one cycle of nivolumab/ipilimumab but ultimately developed grade III checkpoint inhibitors-associated hepatitis. Due to the significantly reduced general condition, no further therapy was administered, and the patient went on to receive best supportive care.

## BRAF pathway and classes of BRAF alterations

**Figure 2:**
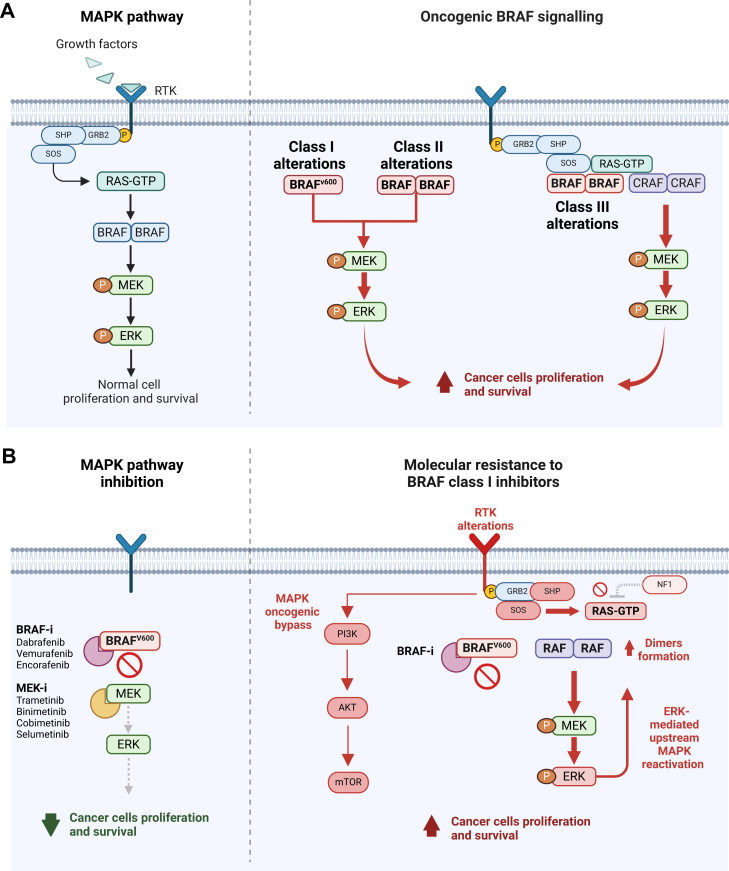
**Physiological and oncogenic signalling in the MAPK pathway.** (A) Under physiological conditions, RAF activity is regulated by RTK-RAS signalling, which can be activated by several factors, including binding of growth factors to RTKs. After acquiring the active conformation, RAF dimers (either containing BRAF, RAF1, or ARAF) mediate the downstream activation of MEK and ERK, leading to normal cell survival and proliferation. Several mechanisms may account for oncogenic BRAF activation, classified within three distinct classes. Class I alterations involve the substitution of valine in position 600 (V600), promoting an active conformation of the kinase leading to the hyperactivity of BRAF-independent monomers. Conversely, class II *BRAF* alterations depend on dimerization to exert their oncogenic function, which similarly to class I alterations do not show dependence on RAS upstream regulation. Lastly, *BRAF* class III alterations do not demonstrate kinase activity (dead-kinase variants), but rather display a higher affinity for RAS, which in turn mediates the formation of BRAF/RAF1 heterodimers ultimately activating the signalling cascade. (B) Mechanism of action of currently approved BRAF plus MEK inhibitors. BRAF inhibitors are capable of binding to BRAF^V600^ monomers, promoting the acquisition of the alpha-C-out inactive structure of the kinase. In addition, MEK allosteric inhibitors mediate the docking of MEK to inactive protein complexes, leading to higher MAPK inhibition, as well as prevent ERK-mediated feedback up-regulation of the pathway, mostly responsible for off-target cutaneous toxicities. Several mechanisms have been described to promote resistance to BRAF class I monomer inhibitors (shown in red). While most mechanisms are driven by ERK-mediated MAPK reactivation, BRAF resistance may also emerge by alterations involving other proteins of the MAPK pathway, as well as genetic aberrations involving parallel signalling cascades, including the PI3K/AKT/mTOR pathway. Created with Biorender.com. ERK, extracellular signal-regulated kinase; MAPK, mitogen-activated protein kinase; RTK, receptor tyrosine kinase.

The RAF family consists of serine-threonine kinases, including ARAF, BRAF and RAF1 (also known as CRAF), which play a pivotal role within the MAPK signalling cascade^26^ (Figure 2A). Under basal conditions, RAF proteins localize within the cytoplasm as auto-inhibited monomers.^26^ Following the engagement by extracellular growth factors with receptor tyrosine kinases (RTKs), adaptor proteins such as Grb2 recruit guanine exchange factors (GEFs), which promote RAS binding to guanosine triphosphate (GTP), subsequently leading to the formation of RAF homo- or heterodimers.^26^^,^^27^ Unmutated RAF proteins undergo allosteric transactivation upon dimerization, a state in which they induce the activation of the downstream signalling pathway by binding to their substrates, mitogen-activated protein kinase kinase 1 and 2 (MEK1/2), which in turn activate extracellular signal-regulated kinases 1 and 2 (ERK1/2), ultimately promoting cell survival and proliferation.^28^

Several types of mutations involving *BRAF* can lead to its oncogenic activation, categorized within three distinct classes (class I-III).

Across all tumour entities, class I alterations account for ∼50% of all *BRAF* alterations,^15^ and consist mostly of substitutions involving the activation loop residue valine 600, most frequently with glutamic acid (V600E) (Figure 2A). The negative charge of E600 mimics the phosphorylation of the adjacent BRAF activation loop residues T599 and S602, an event that is promoted by GTP-loaded RAS upon physiological ERK pathway activation, and switches the kinase domain of wild-type BRAF (BRAF^WT^) and several of its oncogenic counterparts, except BRAF^V600E^, into an active conformation.^29^^,^^30^ Indeed, the mutation-specific salt-bridge formed between K507 and E600 locks the BRAF kinase domain in a constitutively active conformation making T599/S602 phosphorylation superfluous. Similarly, as phosphorylation-induced reorientation of the activation loop relative to other parts of the kinase domain controls this activity switch, other substitutions such as V600K, also induce a highly active oncoprotein. As a result, class I mutations enable BRAF to signal independent of RAS and therefore tumours with these BRAF alterations usually lack mutations in RAS proteins or their activators, at least in a targeted therapy-naïve setting.^15^ Moreover, this RAS independence also means that class I mutants can signal in their monomeric state and are insensitive to ERK-mediated negative feedback phosphorylation loops implicated in dimer disruption.^31^ Nevertheless, work from several laboratories demonstrating that artificially introduced mutations impairing or abrogating the dimerization potential of BRAF^V600E^ hardly affects its transforming properties should not invoke the concept that this oncoprotein always exists as a monomer. In fact, BRAF^V600E^ forms particularly stable homodimers in various cell-free biochemical assays as well as in living cells and is more efficient in phosphorylating MEK as a dimer.^32^^,^^33^ As discussed in the following sections, the fact that BRAF^V600E^ is able to dimerize is of particular relevance from a therapeutic viewpoint as the monomer-selective BRAF inhibitors developed for BRAF^V600E^, vemurafenib, dabrafenib, and encorafenib, are only able to bind to their target in their monomeric state due to a phenomenon of negative allostery.^15^^,^^34^ Thus, any condition increasing dimerization of BRAF^V600E^ will blunt the efficacy of these compounds.

In contrast, class II and III alterations include *BRAF* variants that act in homodimers or heterodimers with RAF1 and ARAF to promote ERK pathway activation^15^^,^^35^ (Figure 2A). Specifically, class II variants contain elevated intrinsic kinase activity compared with BRAF^WT^, while class III alterations display impaired or no intrinsic kinase activity at all. Indeed, the latter mediate their oncogenic activity by forming dimers with catalytically competent RAF proteins such as RAF1 that becomes allosterically transactivated by class III mutants in a RAS-dependent manner.^36^^,^^37^ Consequently, and in stark contrast to class I mutants, which are mutually exclusive with RAS alterations, class III mutants frequently co-occur with gain-of-function mutations in RAS or RTKs, or with loss-of-function mutations in negative regulators such as *NF1*.^35^^,^^38^

## Tackling BRAF alterations and clinical actionability in biliary tract cancer and gastrointestinal malignancies

### Class I mutations

ATP-competitive kinase RAF inhibitors can be grouped into distinct categories reflecting their binding mode to key structural features of the kinase domain such as the αC-helix, R506 in the dimer interface and the orientation of the DFG motif of the activation loop.^39^^,^^40^ As the interplay between activation loop phosphorylation and dimerization affects the orientation of the αC-helix and ultimately switches the kinase domain from the inactive into the active conformation, RAF inhibitors either prefer the inactive or active conformation.^31^ For example, the second-generation BRAF inhibitors vemurafenib, dabrafenib and encorafenib represent so-called type I^1/2^ compounds that bind to BRAF in its monomeric ‘αC-helix-out/DFG-in/R506-in’ conformation. In contrast, sorafenib, the first approved RAF inhibitor, and the more recently developed third-generation inhibitors like naporafenib or tovorafenib represent type II inhibitors binding to RAF proteins in their inactive ‘αC-helix-in/DFG-out/R506-in’ conformation and are able to inhibit and stabilize RAF dimers.^32^ As BRAF^V600E^ is locked in its active conformation by the aforementioned mutation-specific salt-bridge, sorafenib is only active to inhibit this oncoprotein at very high, presumably clinically irrelevant concentrations. Indeed, first clinical trials with the first-generation RAF inhibitor sorafenib, which also inhibits many other kinases,^41^ achieved only limited clinical benefits due to low on-target selectivity and significant off-target toxicities.^42^ In contrast, the second-generation BRAF inhibitors vemurafenib, dabrafenib and encorafenib yielded higher efficacy along with a more favourable safety profile.^43^^,^^44^ Moreover, the combination of second-generation BRAF inhibitors with allosteric MEK inhibitors, such as trametinib, achieved superior pathway inhibition,^45^ together with improved tolerability due to reduced off-target toxicities achieved with the blockade of MEK-ERK feedback signalling, including hyperkeratotic rashes and the potential development of skin tumours.^46^^,^^47^

Following their demonstration of clinical activity in melanoma and non-small-cell lung cancer,^20^^,^^48^^,^^49^ BRAF inhibitors in combination with MEK inhibitors have been tested across solid tumours in basket trial designs.^50^^,^^51^ Specifically in BTC, following case reports showing remarkable responses with the combination of BRAF plus MEK inhibitors,^52^ dabrafenib and trametinib were tested in the single-arm, phase II ROAR trial. Among 43 patients affected by BRAF^V600E^ BTC who progressed on previous chemotherapy regimens, dabrafenib plus trametinib achieved an overall response rate (ORR) of 47% [95% confidence interval (CI) 31% to 62%], with a progression-free survival and OS of 9 months (95% CI 5-10 months) and 14 months (95% CI 10-33 months), respectively. Similarly, in the subprotocol H of the NCI-MATCH trial, three of four patients with BRAF^V600E^ BTC showed a partial response, with one durable response lasting beyond 29 months.^53^ Thus, the combination with MEK inhibitors compared favourably with BRAF inhibition alone, with a phase II basket study of vemurafenib showing one partial response and four stable diseases among eight patients with BRAF^V600E^ BTC.^51^ Despite the limitations of cross-trial comparisons, dabrafenib plus trametinib showed superior activity compared with second-line standard chemotherapy regimens,^54^ establishing itself as the preferred second-line treatment option for BTC carrying *BRAF*^V600E^ alterations.

As opposed to the benefits observed in BTC and other solid tumours, limited activity was seen among colorectal cancer (CRC) in the ROAR trial, in which an ORR of 7% emerged with the use of dabrafenib plus trametinib.^18^ This could be explained by the fact that CRCs, in sharp contrast to other tumour entities, such as melanoma or hairy cell leukaemia, in which BRAF (and MEK) inhibitors show high efficacy, express *a priori* high levels of epidermal growth factor receptor (EGFR). In cells with high ERK activity, the EGFR becomes silenced by multiple negative feedback loops. Consequently, inhibition of the BRAF/MEK/ERK axis results in rapid reactivation of EGFR, which then ensures or even promotes cancer cell proliferation and survival by alternative pathways, e.g. PI3K/AKT, or drives RAF activation trough increased RAS-GTP loading and BRAF inhibitor-assisted paradoxical RAF dimerization.^18^^,^^55^^,^^56^ Accordingly, following the preclinical activity of combined EGFR and BRAF inhibition to suppress tumour growth in CRC models,^55^^,^^56^ the combination of encorafenib and cetuximab, either with or without binimetinib, showed superior efficacy in the phase III BEACON trial, as compared with chemotherapy in pre-treated CRC carrying BRAF^V600E^.^57^ This data ultimately led to the approval of this combination in pre-treated CRC carrying BRAF^V600E^ mutations. Nevertheless, the confirmed response rate was about 25% and mean OS was only extended by ∼4 months, indicating the presence of primary resistance mechanisms in some cases and the rapid onset of acquired drug resistance in the initially responding ones. Mechanistically, this could be explained by preclinical CRC models showing that BRAF (and MEK) inhibitors up-regulate a battery of RTKs not targeted by cetuximab.^58^ Indeed, a clinical case report confirms that RTKs other than EGFR modulate drug responses in BRAF^V600E^-mutant CRCs.^59^ All these data invite for a more comprehensive profiling of RTK expression patterns before and during targeted therapy with the BEACON scheme. Moreover, other BRAF^V600E^-driven cancer entities might also follow the patterns observed in CRC and might require rational combination therapies with RTK blockade as well.

### Class II mutations

BRAF class II mutations produce constitutively active RAS-independent BRAF active dimers, thus resulting in the continuous downstream activation of the ERK pathway (Figure 2A). Compared with BRAF^WT^, class II mutants demonstrate intermediate to high kinase activity and are constituted by mutations primarily located within the activation segment (e.g. K601, L597) or the P-loop (e.g. G464, G469).^60^ Mutations in the activation loop are capable of disrupting interactions with the P-loop, which is responsible for maintaining the kinase in an auto-inhibited state, thus leading to increased intrinsic kinase activity.^38^ Consequently, class II mutations in the P-loop are believed to function in a reciprocal manner to those in the activation segment.^26^ An emerging group of highly active class II mutants is generated by in-frame deletions in exon 12, which has been largely ignored by earlier panel sequencing focusing on the traditional hotspot exons 11 (P-loop) and 15 (activation loop). These mutants, however, are trapped in a constitutively active conformation caused by shortening the segment between the β3 sheet and the aforementioned αC-helix by several, usually five, amino acid residues.^61^^,^^62^ These BRAF^β3-αC^ oncoproteins share their activation mechanism with similar in-frame deletion mutants observed in EGFR and human epidermal growth factor receptor (HER)2^63^ and have been observed in a variety of cancer entities, in particular within KRAS wild-type pancreatic cancers (PMID: 34476331), thereby representing an actionable mutation, for example, with MEK inhibitors. The direct inhibition of these BRAF^β3-αC^ oncoproteins with RAF inhibitors, however, represents an interesting but complex aspect. As the αC-helix is locked into its active ‘in-position’, typical type I^1/2^ compounds like vemurafenib, which bind to their target in the ‘αC-helix-out/DFG-in/R506-in’ conformation, are ineffective, while the ‘αC-helix-in/DFG-out/R506-in’ conformation targeting type II inhibitors, of which none, except sorafenib, has been approved so far, inhibit BRAF^β3-αC^ mutants. Nevertheless, there have been preclinical data and even encouraging case reports showing that pancreatic ductal adenocarcinomas, a melanoma and a Langerhans histiocytosis expressing the particular BRAF^β3-αC^ ΔNVTAP-mutant responded to dabrafenib, either singly or in combination.^62^ Given that dabrafenib is also considered as a type I^1/2^ inhibitor and that others reported only minimal efficacy of dabrafenib against cell lines expressing BRAF^β3-αC^ oncoproteins, Lauinger et al. investigated the dabrafenib sensitivity of these BRAF exon 12 mutants in detail and found that the precise type of in-frame deletion dictates dabrafenib sensitivity, while all of them responded to the clinically trialled type II inhibitor naporafenib.^62^ These data suggest that dabrafenib can be repurposed for these mutants under certain circumstances and provide encouragement for the further clinical development of next-generation type II inhibitors such as naporafenib and tovorafenib.

Besides single-nucleotide variants and small insertions–deletions, class II alterations include BRAF fusion proteins arising from inter- or intrachromosomal rearrangements that translocate the exons encoding the BRAF kinase domain into a variety of other genes.^64^ Despite the diversity of fusion partners, almost all oncogenic fusion partners replace the BRAF exons encoding the RAS-binding (RBD) and cysteine-rich (CRD) domains, which are critical for maintaining BRAF in an auto-inhibited state and need to be displaced by RAS-GTP during physiological BRAF activation in order to allow subsequent dimerization and allosteric transactivation.^65^ As a result, BRAF fusion oncoproteins form very stable and active homodimers and are therefore intrinsically resistant against monomer targeting type I^1/2^ inhibitors like vemurafenib.

Several types of BRAF fusions have been described and detected across various histologies.^64^ Among 97 024 tumour samples, 241 BRAF fusion-positive tumours were detected across 52 histologies together with 82 fusion partners, and which were enriched in pilocytic astrocytomas, gangliogliomas, low-grade neuroepithelial tumours and acinar cell carcinoma of the pancreas.^66^ In the same study, among 24 patients, treatment with MEK inhibitors with or without BRAF monomer inhibitors resulted in 2 partial responses, 11 stable disease and 7 primary progressive diseases, showcasing preliminary evidence of MEK inhibition as a valuable treatment strategy to tackle BRAF fusions. Moreover, in a single-arm, phase II study, among 137 paediatric patients with low-grade glioma, of whom 74% were carrying BRAF fusions, durable responses were observed with the class II RAF inhibitor tovorafenib, showing an ORR of 67% and a median duration of response of 16.6 months, which were consistent among tumours pre-treated with BRAF/MEK inhibition, and which led to the approval of tovorafenib for refractory *BRAF*-altered paediatric low-grade glioma.^67^

### Class III mutations

Class III *BRAF* mutations exhibit low or no kinase activity at all, for example due to somatic mutation of the codon for D594 that plays an essential role in catalysis. Biochemical and genetic approaches in mice revealed that these BRAF mutants rely for their oncogenic role on upstream RAS activation and a catalytically competent dimerization partner that becomes allosterically transactivated by the class III mutant in a similar way as also kinase-inhibited BRAF can trigger paradoxical ERK pathway activation by binding to a drug-free protomer.^35^, ^36^, ^37^^,^^68^ Thus, class III BRAF proteins do not directly phosphorylate MEK, but trigger MEK/ERK phosphorylation through their increased ability to bind and activate RAF1^69^ (Figure 2A).

As a result, tumours with class III mutations cannot be treated with BRAF^V600E^-selective compounds but may respond to inhibitors that target upstream components of the pathway, such as RTKs inhibitors or direct RAS inhibitors. Growing evidence indicates that patients with class III *BRAF*-mutant CRC may benefit from a combination of EGFR blockade and chemotherapy, without the need for BRAF inhibitors, due to their reliance on the upstream *RTKs* activity for their oncogenic role.^35^^,^^70^ However, there are also strategies directly inhibiting the heterodimer between RAF1 and a kinase-dead BRAF oncoprotein. For example, sorafenib, which targets RAF dimers and was originally developed as a RAF1 inhibitor, blunts the paradoxical MEK/ERK activation potential of kinase-dead BRAF in melanoma and CRC cell line models.^37^^,^^71^ These insights were used to treat a patient with a standard treatment-refractory melanoma carrying a BRAF^D594G^ mutation with a combination of sorafenib and trametinib, yielding a response for almost 5 months.^72^ More recently, a preclinical study showed that encorafenib, which despite being a type I^1/2^ inhibitor is still able to block BRAF dimers at higher concentrations,^34^ can be used in combination with binimetinib to impair oncogenic signalling in melanoma lines by class III mutants.^73^ As tumours with class III *BRAF* mutations usually contain elevated RAS-GTP levels and as type I^1/2^ compounds like encorafenib can unfold paradoxical activity, such an approach might require careful and tight control intervals in a clinical setting.

## Molecular mechanisms of resistance to BRAF^V600^ inhibitors

Despite the remarkable results achieved with BRAF class I inhibitors, several unmet clinical challenges remain. Although durable responses are observed, resistance ultimately develops in most BRAF^V600E^ tumours exposed to BRAF and MEK inhibitors. Different mechanisms have been described to promote resistance to second-generation BRAF inhibitors, occurring either through alterations in the *MAPK* pathway or by the activation of alternative signalling pathways.

In the former group, as opposed to other oncogenic kinases, gatekeeper mutations impeding inhibitor access to the BRAF ATP-binding pocket are particularly uncommon.^74^, ^75^, ^76^ Rather, the primary mechanism of molecular resistance is represented by the increased formation of RAF dimers,^75^, ^76^, ^77^ which can be promoted by several mechanisms. Particularly, alterations can directly involve BRAF through gene amplifications or splice site variants deleting the exons for the auto-inhibitory moiety consisting of RBD and CRD, leading to the formation of BRAF homo- or heterodimers by the same mechanism as described in the preceding text for BRAF fusion oncoproteins.^74^^,^^76^ Alternatively, molecular alterations can involve upstream MAPK regulators (i.e. RTK, KRAS, NRAS, NF1)^78^ or downstream effectors, mostly mediated by ERK-mediated feedback reactivation of RTKs, which ultimately promote *RAF* dimer formation.^75^^,^^76^^,^^79^^,^^80^ In addition, BRAF monomer inhibitors themselves have been described to promote the process of RAF dimerization.^81^^,^^82^ In detail, while the binding of selective inhibitors to BRAF^MUT^ protomers induces an inactive αC-out conformation of the activation loop and kinase suppression, the same mechanism can promote the active αC-in conformation by wild-type RAF drug-unbound monomers, ultimately leading to MAPK signalling reactivation in the so-called ‘RAF inhibition paradox’.^81^^,^^82^

Another mechanism promoting resistance to BRAF^V600^ inhibition consists of *BRAF* gene amplifications, which have been described both as an acquired mechanism and as well as a primary resistance mechanism potentially driven by tumour subclones carrying baseline BRAF copy number gains.^83^^,^^84^ Interestingly, in an exploratory analysis of the ROAR trial involving 19 patients with BTC, gene expression levels of key members of the MAPK pathway (BRAF, CRAF, HRAS, KRAS, MAP3K8, NRAS, and NF1) demonstrated an association with clinical benefits from BRAF^V600^ inhibition.^9^ In detail, among two patients showing primary resistance, higher expression levels of MAPK genes were observed compared with cases showing stable disease (*n* = 9) or partial response (*n* = 8). These again show that higher expression of the MAPK genes are primary mechanisms of resistance.

Alternatively, resistance to BRAF inhibitors can emerge by reducing tumour cell dependency of the MAPK pathway by the concurrent activation of alternative signalling cascades, including the PI3K/AKT/mTOR pathway,^85^, ^86^, ^87^ cell-cycle-affecting alterations involving *CCND1* and deletions of CDK2NA^88^, ^89^, ^90^-enhanced RTK signalling (e.g. MET, PDGFRβ, IGF1R, HER1 among others),^91^, ^92^, ^93^ or by downstream transcriptional aberrations mediated by the WNT/β-catenin and HIPPO signalling cascades.^94^, ^95^, ^96^ The latter is able to determine the up-regulation of RTK*s* such as HER2 and HER3 or induce the up-regulation of anti-apoptotic proteins (e.g. BCL2, BCL-X_L_) mediated by ERK, which contribute to anti-BRAF resistance.^96^^,^^97^

Therefore, several molecular mechanisms are responsible for molecular resistance to BRAF inhibition, uncovering the complex genomic interplay capable of mediating clinical resistance to currently approved BRAF and MEK inhibitors.

## Emerging treatment strategies to overcome molecular resistance

Considering the wide range of molecular mechanisms possibly governing resistance to BRAF inhibitors, several treatment strategies and novel compounds are being currently developed and investigated across preclinical and early-phase clinical studies.

Given the key role of RAF dimers in overcoming BRAF^V600^ monomer inhibition, novel RAF inhibitors capable of preventing the formation of RAF dimers have been designed. Among them, the so-called RAF monodimer inhibitors have shown the ability to inhibit BRAF monomers while inducing conformational changes within the *RAF* dimerization domain, which can prevent RAF molecule dimerization.^15^^,^^98^ However, the inhibition of RAF dimers containing also RAF1 and ARAF alone has been associated with additional toxicity and thus inferior tolerability,^77^^,^^99^, ^100^, ^101^ which prompted the development of selective BRAF dimer inhibitors, which are able to spare the inhibition of RAF1 and ARAF containing homodimers, resulting in reduced off-target toxicities and better tolerability.^15^ Among these, plixorafenib and ARRY-440 have both demonstrated preliminary signals of activity among class I BRAF^MUT^ tumours previously exposed to second-generation BRAF inhibitors, as well as for non-class I alterations in early-phase clinical trials.^77^^,^^102^

In addition to ATP-competitive kinase inhibitors, allosteric BRAF inhibitors are currently under development, including drugs targeting MEK and promoting an inactive BRAF-MEK complex leading to a decreased downstream signalling, as well as small peptides able to interfere with MAPK signalling by binding to the RAF dimer interface.^103^^,^^104^ Of note, the MEK-targeting molecular glue NST628, which is capable of promoting the formation of MEK:A/B/C-RAF-inactive complexes, has demonstrated encouraging preclinical activity among all classes of *BRAF* alterations, together with a synergistic effect of its combination with sotorasib in both RAS- and RAF-driven cancer models.^105^

Besides novel compounds, some multikinase inhibitors with relatively low selectivity for BRAF such as ponatinib, or type I BRAF inhibitors able to inhibit other kinases at higher concentrations such as vemurafenib, have been described to inhibit RAF dimer formation by structural alteration of the kinase αC-helix loop, and thus to potentially act also on BRAF class II and III alterations.^34^ Regardless, limited activity was observed with vemurafenib in the phase II TAPUR trial, in which among 28 patients affected by different tumours carrying several types of BRAF^MUT^ (*n* = 3 amplifications, *n* = 5 class I, *n* = 10 class II, *n* = 8 class III), only two partial responses were recorded, with a disease control rate of 21%.^106^

In addition to targeting BRAF, combination strategies targeting either different MAPK components or parallel signalling cascades are currently under investigation. Several efforts are underway to target multiple nodes in the MAPK pathway (vertical pathway inhibition), either by targeting RAS regulators (e.g. SOS1, SHP2)^107^^,^^108^ or ERK. Of note, the first-in-class ERK inhibitor ulixertinib showed preliminary activity against all BRAF^MUT^ classes in patients affected by different solid tumours.^109^

Alternatively, to overcome the oncogenic bypass of the MAPK pathway through the activation of parallel signalling cascades, combinatorial strategies are currently being investigated in preclinical models or early-phase clinical trials, involving the use of compounds targeting the PI3K/AKT/mTOR pathway,^110^ proteins involved in cell cycle regulation with CDK4/6 inhibitors,^88^^,^^111^ RTKs (e.g. HER2, HER3),^112^^,^^113^ anti-apoptotic proteins up-regulated by ERK and the HIPPO pathway,^97^^,^^114^, ^115^, ^116^ or FAK up-regulation mediated by WNT signalling particularly among CRCs.^94^

Accordingly, the several mechanisms potentially governing resistance to BRAF inhibitors represent the rationale for testing multiple strategies to tackle acquired molecular resistance to class I BRAF inhibitors, with novel RAF dimerization inhibitors showing preliminary activity also towards non-class I alterations, and with combination strategies targeting the MAPK or parallel pathways which should be further implemented in a biomarker-driven manner.

## Concluding remarks

BRAF class I inhibitors have demonstrated remarkable clinical benefits across solid tumours carrying BRAF^V600E^ alterations, ultimately leading to the FDA tumour-agnostic approval of dabrafenib and trametinib for solid tumours and to the authorization of encorafenib plus cetuximab for pre-treated, BRAF^V600E^-mutated CRC, owing to the intrinsic property of EGFR signalling to overcome BRAF inhibition. However, several unmet biological and clinical challenges persist in targeting BRAF, primarily involving the development of resistance to BRAF plus MEK inhibitors mostly mediated by *ERK*-driven up-regulation of RAF dimerization. The several potential mechanisms underlying resistance to BRAF inhibition demand exploring diverse treatment strategies, including novel RAF monomer–dimer inhibitors, which may also be effective against non-class I mutations, or combination strategies for tumours showing MAPK oncogenic bypass. Despite these advances, better tolerability of novel RAF inhibitors must be achieved, as well as developing inhibitors with reduced off-target toxicities enabling the use of tolerated combinatorial treatment strategies. Furthermore, elucidating potential histology-specific resistance factors remains essential to optimize treatment strategies in tumours showing resistance to BRAF inhibitors, and to further achieve better clinical outcomes by means of precision oncology in human cancers driven by oncogenic *BRAF* alterations.

## Funding

None declared.
